# Localization of coronary bypass targets in hard-to-see coronary arteries

**DOI:** 10.1186/s13019-023-02399-8

**Published:** 2023-10-12

**Authors:** Rabin Gerrah, Kristin Lipe, Gus J. Vlahakes

**Affiliations:** 1 Department of Cardiothoracic Surgery, Stanford University, Falk Bldg. 300 Pasteur Drive, Stanford, CA 94305-5407 USA; 2 Department of Surgery, Good Samaritan Medical Center, Corvallis, OR USA; 3 Division of Cardiac Surgery, Harvard Medical School and Massachusetts General Hospital, Boston, MA USA

**Keywords:** Coronary artery disease, Bypass grafting

## Abstract

**Background:**

Precise identification of coronary arteries and selection of anastomotic sites are critical stages of coronary bypass surgery. Visualization of coronary arteries is occasionally challenging when the heart is covered with a thick layer of fat or scar tissue. In this paper, we review the methods to localize the coronary arteries during coronary surgery.

**Methods:**

Prior publications were searched to summarize all available methods for localization of coronary arteries during coronary surgery.

**Results:**

Five clinically recognized and three experimental techniques from the literature review are reviewed and summarized.

**Conclusions:**

Knowledge of various techniques of coronary artery identification in hard-to-see coronary arteries is an important asset in coronary surgery and especially useful during the most critical option of the most common heart surgery.

## Introduction

**Fig. 1:**
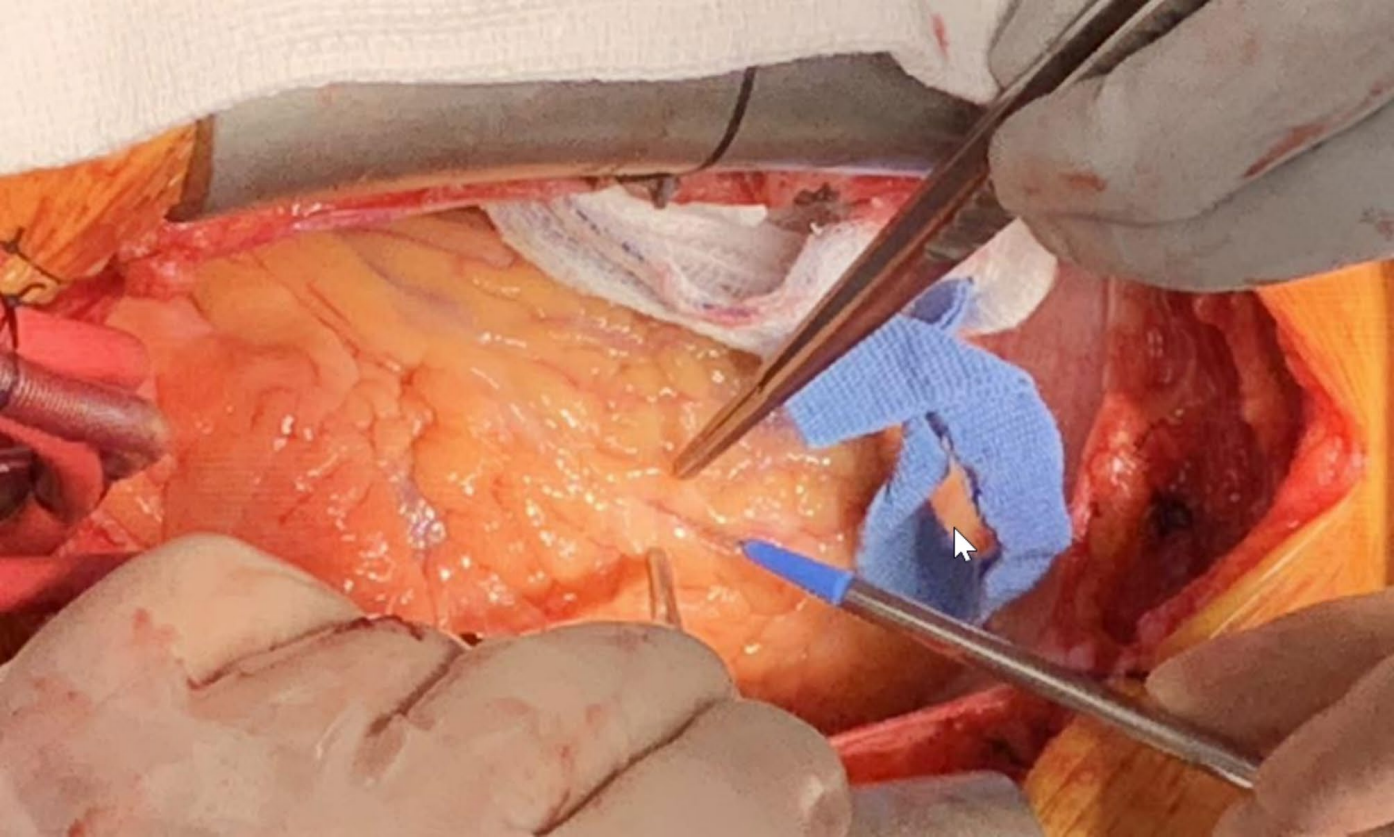
Exploring a coronary artery in the fat covered heart after identification of severely calcified coronary artery by palpation

During coronary artery bypass grafting, the epicardial coronary arteries (CA) are identified, exposed, and opened where the bypass grafts will be anastomosed (Fig. 1). Optimal coronary anastomosis construction and the outcome of coronary bypass surgery depends on identification of the correct vessel and precise arteriotomy, avoiding angled, transverse, or otherwise imprecise anastomoses. Most often, the epicardial CA are clearly visible and can be easily identified through simple observation and palpation. In some instances, however, identification of the coronary arteries becomes challenging when these vessels are intramyocardial or covered with a dense layer of adipose tissue or scar tissue such as in reoperative surgery. Localization of hard-to-see coronary arteries becomes even more challenging following aortic cross-clamping and cardioplegia administration, which results in the absence of color differentiation afforded by arterial and venous coronary flow. Attempts to expose an artery in adipose tissue further complicate the exposure of the artery with consequent bleeding from the dissection needed to find the desired anastomotic site. The process becomes further complicated when a coronary vein is running in the assumed arterial path and is erroneously identified as a bypass target.

Hard-to-identify coronary arteries are encountered more frequently due to the increasing prevalence of obesity with increased epicardial fat and the increased incidence of reoperative surgery. In addition, with certain comorbidities such as diabetes mellitus, epicardial fat may be significantly increased [1]. In general, patients with coronary disease have a high propensity for larger volume epicardial fat as there is an established correlation between epicardial adipose tissue thickness and the severity of coronary disease [2]. Most of the epicardial fat is accumulated and located in the atrioventricular and interventricular grooves, as well as along major branches of the coronary arteries [3].

In this paper, we review not only the practical solutions, but also novel experimental methods to identify the CAs when they cannot be easily visualized during coronary surgery.

## Methods

In this article, the literature review as well as personal experience in the identification of coronary arteries in hard- to- see coronary surgery cases are summarized. Advantages and disadvantages of each technique are discussed in detail. The purpose of this review is to provide an organized guide to currently available clinical methods as well as experimental methods that might be thought provoking and might generate newer ideas in the future.

### Palpation of the calcified vessel

Diseased coronary arteries that require revascularization may be calcified. Palpation of a calcified vessel might give some clue or direction towards the location of the vessel, and it will also help to identify the area with less calcification, more appropriate for anastomosis. Although this is the easiest method, the low resolution of human tactile sense in a gloved hand and the lack of calcification at a desired anastomotic site make this method far from being an optimal solution for finding an anastomotic site which is not visible. Palpation of the CA is especially useful techniques in previously stented CA. The rigid stent can be easily palpated in the CA and its trajectory can guide in determination of the CA route when it is not easily visible.

### Tracking distal arterial branches towards a proximal anastomotic site: role of retrograde probing

It is sometimes possible to track a smaller vessel in a known distribution area proximally towards the larger part of the vessel covered in adipose or scar tissue. This technique still relies relying on palpation, which is particularly useful for localizing obtuse marginal and right CA branches.

Retrograde probing is a technique useful for localizing the mid and proximal left anterior descending (LAD) CA. At this distal location, the vessel is more likely to be visible on the surface of the heart. If the LAD is small in this location and terminates at the apex, it can be opened and a 0.5-1.0 atraumatic probe can be inserted and directly proximally to be located by palpation, thus providing localization to minimize the amount of dissection needed to expose it. In a very small, terminating LAD, the insertion site can be ligated without consequence.

If the distal LAD is large and wraps around the apex of the heart to supply the distal interior wall, the same technique can be used with some modifications. A transverse arteriotomy can be made approximately one-fourth to one-third of the circumference of the LAD in this location. An atraumatic probe can then be advanced proximally to localize a proximal site on the LAD. However, it is essential that the distal arteriotomy not be extended by inadvertent tearing or traction from the probe. When the proximal LAD is identified, the probe can be withdrawn and the short transverse arteriotomy closed with interrupted 8 − 0 or 7 − 0 polypropylene sutures.

### Antegrade probing of the vessel

**Fig. 2:**
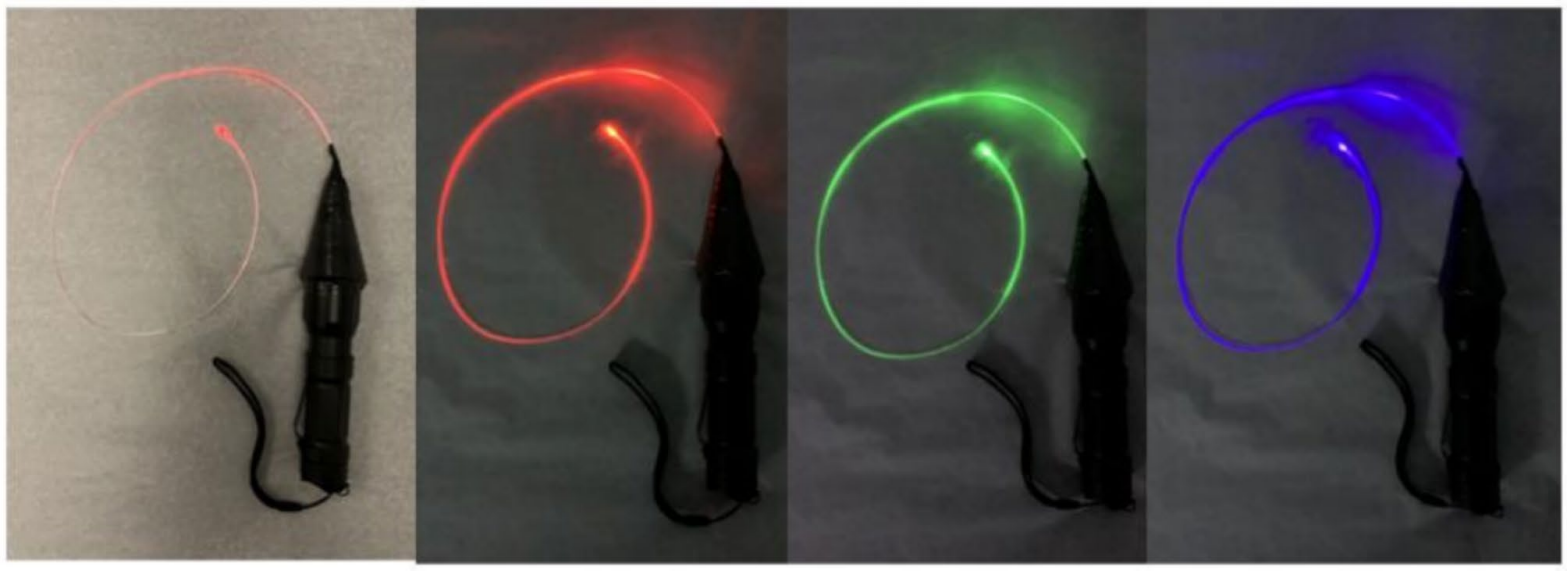
Assembly of an optical fiber on a flashlight with various illumination options shown in dimmed ambient light

If previous techniques fail, antegrade probing is another option. This is certainly easily done if there is a concomitant aortic valve replacement, but if not and if the desired coronary artery target cannot be found, the time needed to make a short arteriotomy is certainly justified. A probe or a guidewire is advanced under direct vision into a coronary vessel to be grafted (Fig. 2). Presence of the probe or guidewire in the CA can be palpated, particularly with gentle motion of the probe or guidewire. Clearly, a totally occluded CA obviates this option.

### Thermal imaging

**Fig. 3:**
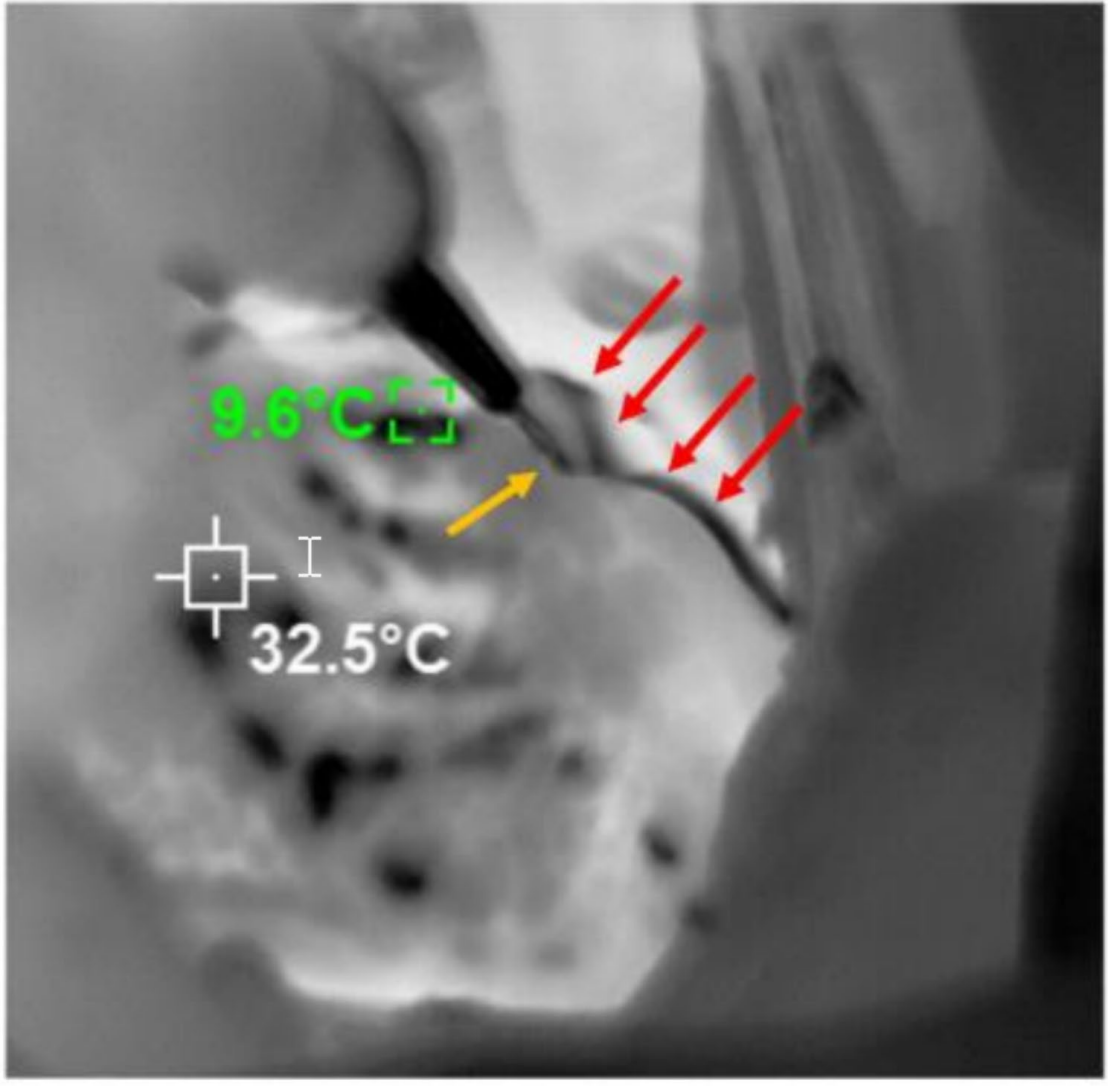
Thermal imaging of the left anterior descending coronary artery immediately after infusion of cold cardioplegia. The red arrows show the track of the artery which was not visible under the thick fat pad. The yellow arrow indicates the tips of a Gerald forceps held in surgeon’s hand pointing toward the course of the artery. The imaging should be done fast with initiation of the cardioplegia solution so minimal amount of cold solution reaches the coronary vein

Thermal imaging of the coronary arteries has been used to ensure universal perfusion of the cardioplegia solution and myocardial cooling and for determination of graft patency [4]. This technique based on the principle of thermal differential in the tissue can be utilized to identify the coronary arteries. A CA can be visualized by infusion of a different temperature solution in the coronary arteries, which behaves as a contrast media and visualizes the CAs like in coronary angiography, hence the term thermal coronary angiography. For CA tracking, a handheld thermal imaging camera with a resolution of 384 × 288 pixels and a frame rate of 25 Hz (HTI-Xintai: www.hti-instrument.com) is pointed towards the exposed surface with the CA of interest. Cold antegrade cardioplegia solution is infused through the usual cardioplegia needle. As the different temperature cardioplegia flows in the CA, it will visualize the path of the flow and the course of the CA in real time (Fig. 3). Because of the direct visualization this technique provides, and being less invasive, it seems to be useful in most cases.

### Doppler ultrasound localization

Doppler ultrasonography using a linear array transducer, with a high frequency imaging system (e.g., 7.5 MHz or higher) can clearly visualize a CA on the beating heart and during cardioplegia infusion. A short or long axis view of the vessel may significantly help in selection the best arteriotomy site on the artery for grafting. Note that depending on the frequency of the ultrasound probe, a “standoff” may be needed to provide imaging close to the surface of the heart.

High frequency epicardial ultrasound has been used to locate coronary arteries and branches, and this technique has been also useful to differentiate arteries and veins by using Doppler imaging [5]. Ultrafast Doppler coronary angiography can visualize coronary vessels as small as 100 micrometers as well as flow quantification of coronary blood flow [6].

### Intraoperative cineangiography

Angiography is considered a gold standard technique for vascular studies. Intraoperative angiography has been used to evaluate vein graft patency during coronary bypass surgery [7]. Although a very useful technique to track the coronary path, determination of the best anastomosis spot and also a useful tool in assessment of the anastomosis quality, the widespread use of intraoperative cineangiography is limited as these imaging requires x-ray systems, available only in the hybrid operating room, administration of intravenous contrast, availability of additional technologist. Unless preoperatively planned and scheduled, this modality is less useful for intraoperative localization of CA.

### Experimental techniques

#### Optical fiber probes

**Fig. 4:**
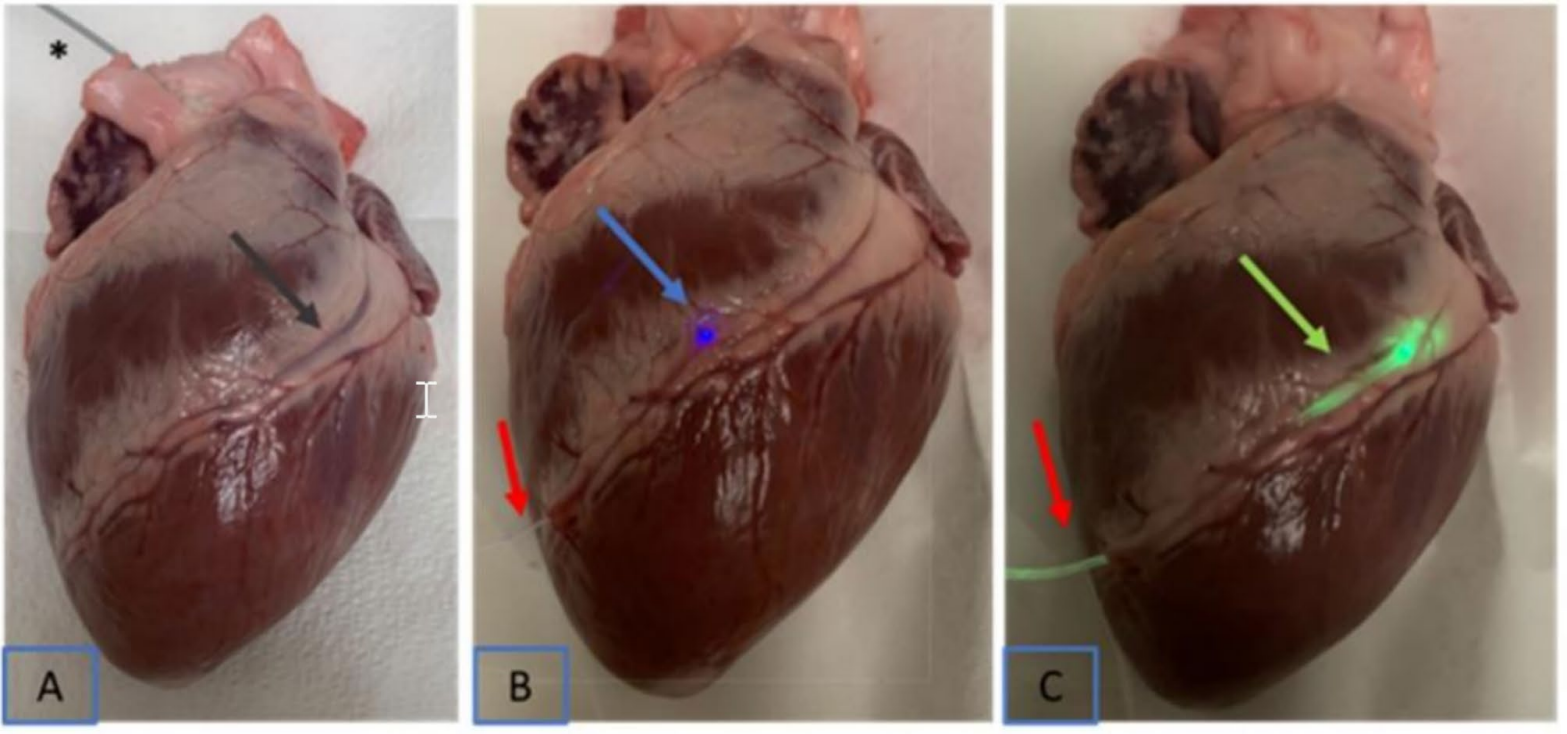
Three visualization techniques in a fresh porcine heart. Identification of the coronary artery by placing a proximal guidewire (**A**) probe in the coronary artery through the left coronary ostium via the aorta (asterisk and grey arrow) and an optical fiber through distal left anterior descending coronary artery, red arrows (**B**, **C**): (**B**) visualization by an end-emission optical probe using blue light, (blue arrow), (**C**) by a side-emission optical probe with green light, (green arrow)

Similar to antegrade and retrograde mechanical probing of coronary arteries, a light transmitting fiber can be used to transilluminate a CA. Provided in in 0.03’’ diameter as either end-emission or side-emission, the optical fiber illuminates the end position of the fiber in the vessel or the track of the CA, respectively (Fig. 4B C). Such a device can be assembled from readily available materials. A PMMA (Polymethyl methacrylate) optical fiber that is generally used for decoration purposes {www.AMAZON.com: PMMA Plastic Fiber Optic End Glow Cable 0.03in/0.75 mm}is attached to a bright light source, such as a non-proprietary flashlight {LUMENSHOOTER 4 Color in 1 Multi-Color Tactical Flashlight Torch} that provides intense illumination in a selectable color that will be emitted from the tip of the fiber. The fiber is long, and it can be sterilized, and one end can be handed off to the back table to be connected to a light source with different colors or optionally connected with a custom-made connector to the headlight light source available in every cardiac operating room.

#### Tracking conductivity

**Fig. 5:**
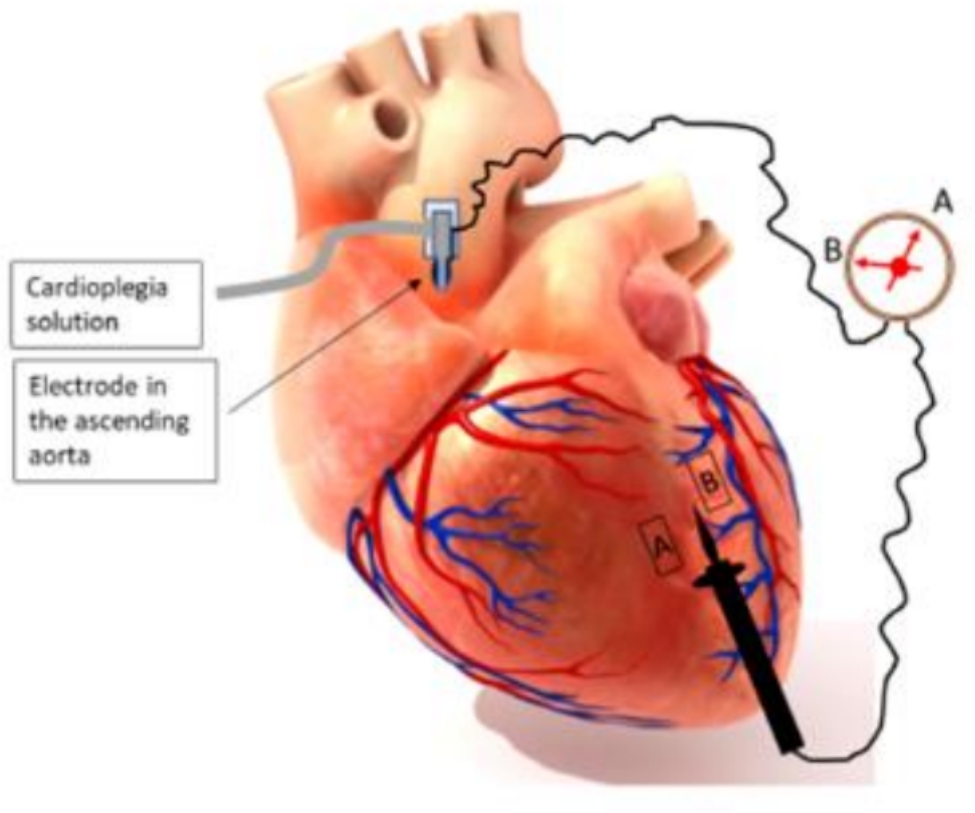
Measurement of differential electrical impedance on the heart surface. As the probe approaches the path of the coronary artery (flowing conductive solution), from point A to point B, the impedance will drop. Since the measurement is subject to factors such tissue thickness, the impedance measurements not in absolute but in relative values with the variable of interest being the lowest impedance, correlating to closest point to artery carrying the solution. Since both artery and vein carry the solution, the impedance reading might not differentiate between a vein and an artery. A multi-array patch electrode might be needed for instantaneous reading of the impedance (before the solution reached the vein) or alternatively the differentiation might be facilitated by combination of a dye to this technique

Similar to the concept of thermal imaging, perfusion of electrolyte rich isotonic content solution such as cardioplegia solution, which has high electrically conductive properties in coronary arteries, modifies the electrical impedance of the myocardial tissue. The tissue along the vessel carrying the conductive substance (closest to the vessel wall) will show the least impedance. The differential electrical impedance is measured between the point of reference (such as a needle attached to a wire or just a wire in the aortic root), and a handheld electrode on the surface of the heart while the cardioplegia solution is in constant flow, Fig. 5. A consumer- grade highly sensitivity multimeter connected to a sterile cable can be used for detection of the point with the least resistance to electrical flow, and hence, closest to the target artery.

#### Color injection

**Fig. 6:**
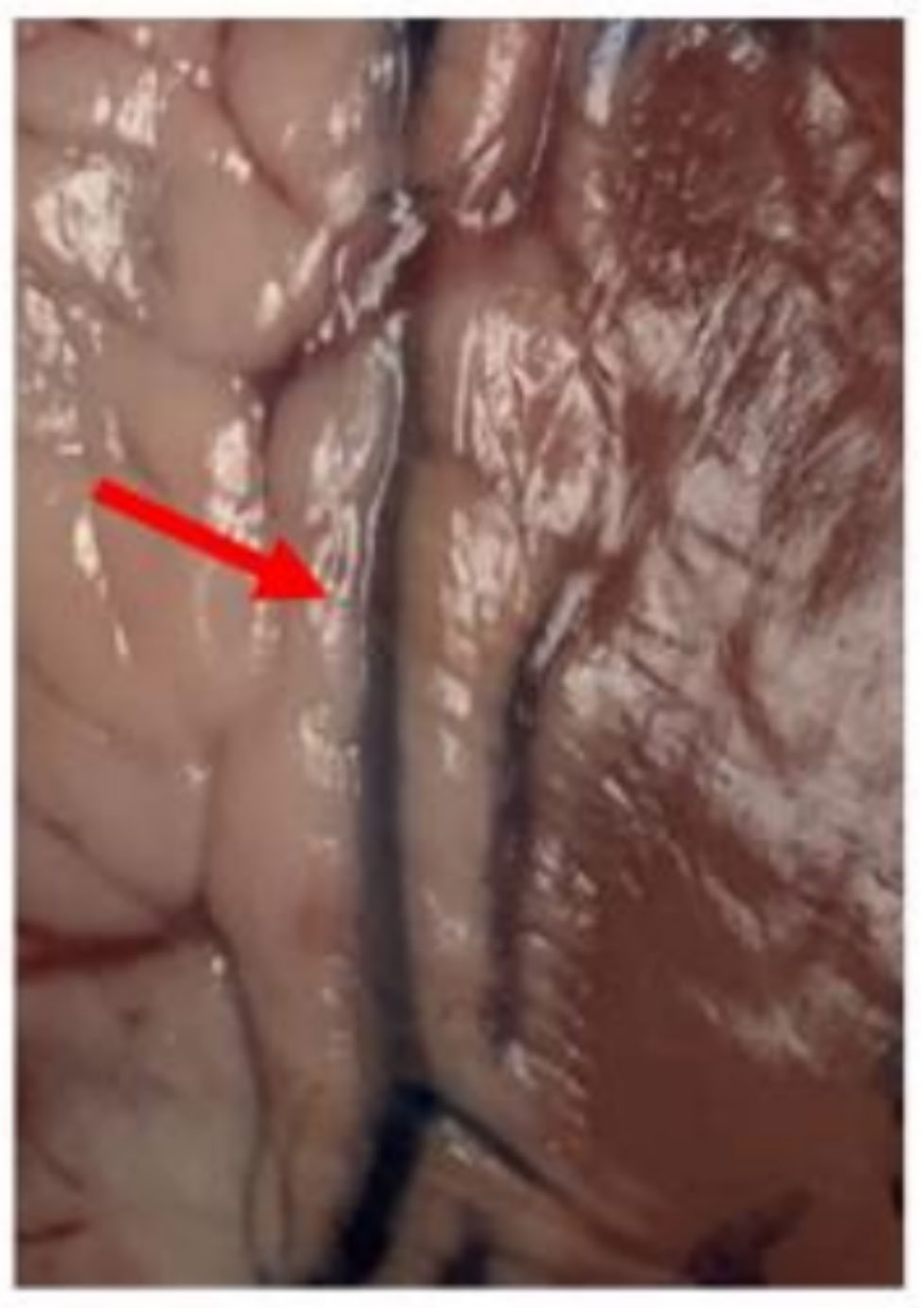
Close-up view of a coronary artery visualized by injection of methylene blue dye in the coronary ostium (red arrow). The injected artery is clearly visualized distinct from other arteries and also from the coronary veins

Diagnostic dyes are commonly used in clinical applications: indocyanine green (Spy Agent Green) is used in fluorescence imaging for angiographic determination of cardiac output and hepatic blood flow. Intraoperative laser angiography using Spy system is a useful method in evaluation of blood flow and perfusion, which can be easily used for localization of the CA [8]. Methylene blue is used to treat methemoglobinemia and in the treatment of vasoplegic syndrome and can be easily tracked under normal light in the CA. Fluorescein has been used to visualize residual muscular ventricular septal defects [9] and fluorescein angiography is mostly to detect retinal vasculature. Use of colors has been reported as a technique for surgical planning in congenital heart surgery [10] when incisions in the ventricles might damage an intramyocardial coronary artery. The same technique can be used in coronary surgery (Fig. 6). Once the color has identified the arterial target as distinct from a coronary vein, the coronary circulation should be flushed with cardioplegia to wash the methylene blue from the anastomotic site before arteriotomy is made.

#### Comment

In this paper, we have reviewed the most common methods to identify the CA for bypass grafting during coronary surgery. In our experience, approximately 10% of coronary bypass cases have CAs that are not easily visible and require more attention with localization of the CA and selection of optimal bypass spot. Of all methods described in this paper, authors use palpation, tracking, retrograde probing and rarely thermal imaging in selected cases with hard-to-see CA. While some localization techniques are more invasive and require incisions or opening, some others are less invasive and easier to employ. Localization of the embedded CA becomes even more challenging in off-pump coronary bypass surgery and in these scenarios less invasive techniques are more appropriate. Of all techniques described in this paper, the least invasive is probably the thermal imaging, which, due to the super-fast technology improvement, is feasible in every operating room. Familiarity with all these techniques is crucial during the most critical option of the most common heart surgery.

## Data Availability

NA.
